# Prevalence and antimicrobial resistance trends of ESKAPEE pathogens in clinical isolates from Hyderabad, Pakistan: a cross-sectional study

**DOI:** 10.1016/j.ijregi.2026.100950

**Published:** 2026-07-18

**Authors:** Muhammad Aqif Ilyas, Muhammad Sameer Hashmi

**Affiliations:** 1 Institute of Microbiology, University of Sindh, Jamshoro, Pakistan; 2 Department of Physiology and Medical Laboratory Technology, University of Sindh, Jamshoro, Pakistan

**Keywords:** ESKAPEE pathogens, Antimicrobial resistance, Multidrug-resistant organisms, Nosocomial infections, Pakistan

## Abstract

**Objectives:**

Antimicrobial resistance is a major global health threat that limits treatment options and increases morbidity and mortality. ESKAPEE pathogens are prioritized by the World Health Organization and the Centers for Disease Control and Prevention because of their role in nosocomial infections and their ability to evade antimicrobials. This study aimed to determine the prevalence and antimicrobial resistance patterns of ESKAPEE pathogens in Hyderabad, Pakistan.

**Methods:**

A cross-sectional study was conducted from August 2023 to August 2024 at the University of Sindh. A total of 1072 clinical specimens were processed for bacterial isolation, identification, and antimicrobial susceptibility testing following Clinical and Laboratory Standards Institute and European Committee on Antimicrobial Susceptibility Testing guidelines. Isolates were classified as multidrug-resistant, extensively drug-resistant, or possible pandrug-resistant using the Magiorakos et al. criteria and the Centers for Disease Control and Prevention/Infectious Diseases Society of America definitions.

**Results:**

Among positive cultures, ESKAPEE pathogens accounted for 82.1% of isolates. *Escherichia coli* was the most prevalent (49.8%), followed by *Klebsiella pneumoniae* (13.2%), *Acinetobacter baumannii* (9.7%), *Pseudomonas aeruginosa* (9.3%), *Enterococcus* spp. (8.3%), *Staphylococcus aureus* (8.0%), and *Enterobacter* spp. (1.7%). High resistance was observed to β-lactams, cephalosporins, and fluoroquinolones. Carbapenem resistance ranged from 11.1% to 25.7% in *E. coli*, 29.0% to 39.5% in *K. pneumoniae*, 48.2% in *P. aeruginosa*, and 60.7% to 64.3% in *A. baumannii*.

**Conclusion:**

The high burden of multidrug-resistant and extensively drug-resistant ESKAPEE pathogens highlights the urgent need for strengthened antimicrobial stewardship, continuous surveillance, and rigorous infection-control strategies.

## Highlights

• ESKAPEE pathogens accounted for 82.1% of culture-positive clinical isolates.
• *Escherichia coli* and *Klebsiella pneumoniae* were the predominant resistant pathogens.
• High resistance was observed to β-lactams, cephalosporins, and fluoroquinolones.
• Substantial carbapenem resistance occurred in major Gram-negative pathogens.
• Strengthened antimicrobial stewardship and infection control are urgently needed.

## Graphical Abstract

**Figure:**
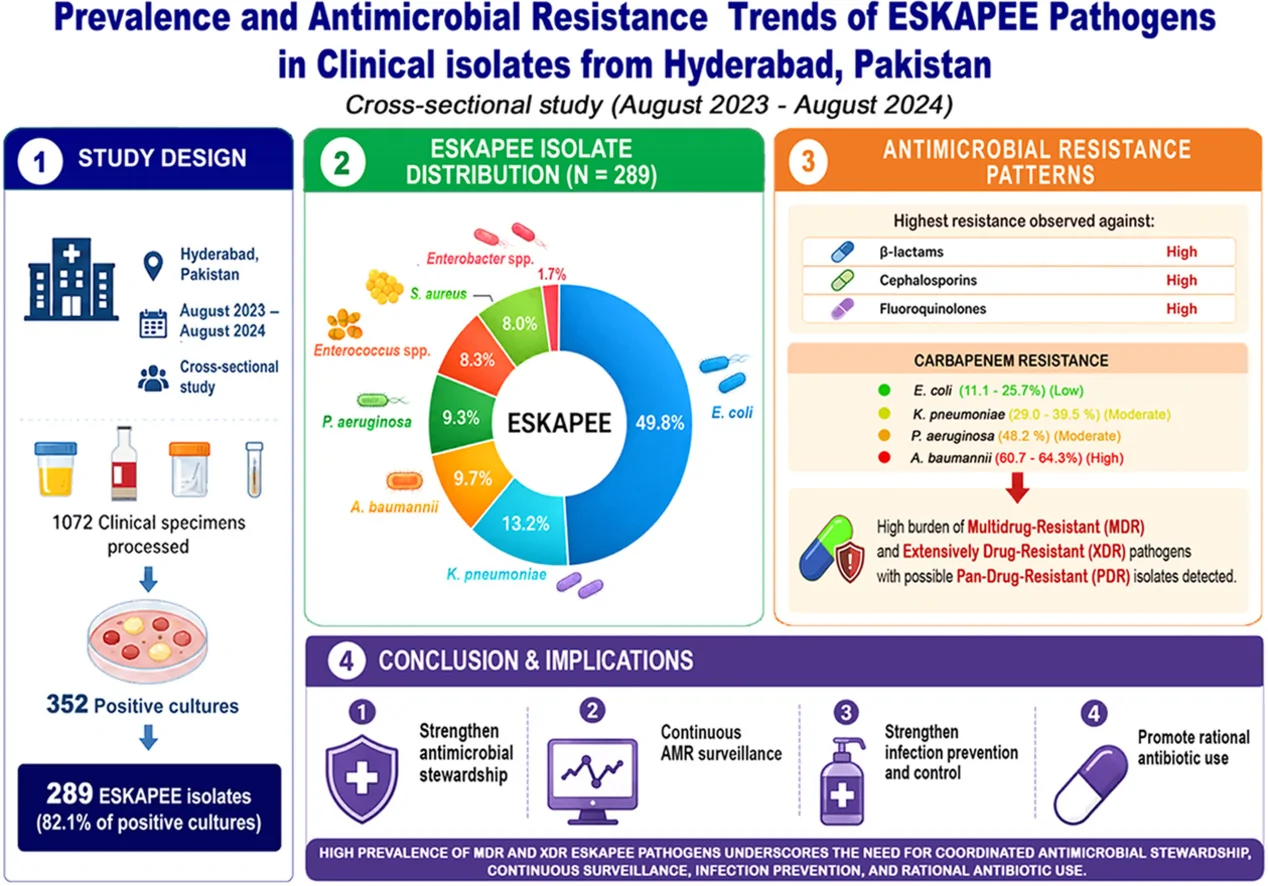

## Introduction

The global spread of multidrug-resistant bacteria has increased hospital mortality and reduced effective treatment options, making antimicrobial resistance (AMR) a major public health threat. In 2019, an estimated 1.27 million deaths were directly attributable to antibiotic-resistant bacterial infections, while approximately 4.95 million deaths were associated with antimicrobial resistance overall [1]. By 2050, antimicrobial resistance has been projected to cause up to 10 million deaths annually if effective interventions are not implemented [2]. More recent modeling suggests this early projection is proving accurate rather than overstated, estimating that AMR could account for approximately 1.91 million attributable and 8.22 million associated deaths each year by 2050, with South Asia projected to bear one of the highest regional burdens [3]. As part of this region, Pakistan is likely to face a substantial AMR burden because of challenges including inappropriate antibiotic use, surveillance gaps, and inadequate antimicrobial stewardship [1,3].

The ESKAPE pathogens (*Enterococcus faecium, Staphylococcus aureus, Klebsiella pneumoniae, Acinetobacter baumannii, Pseudomonas aeruginosa*, and *Enterobacter* spp.) are highly virulent, multidrug-resistant bacteria that pose serious health care challenges and contribute substantially to morbidity, mortality, and health care costs [4]. The group is sometimes expanded to ESKAPEE to include *Escherichia coli* [5]. Because of their ability to evade commonly used antibiotics, these Gram-positive and Gram-negative pathogens are leading causes of life-threatening nosocomial infections, particularly in immunocompromised and critically ill patients [6].

The ESKAPEE pathogens employ multiple mechanisms to evade antimicrobial action, including drug inactivation, target modification, reduced intracellular drug accumulation, and biofilm formation. Drug inactivation occurs through enzymes such as β-lactamases, aminoglycoside-modifying enzymes, and chloramphenicol acetyltransferases. Target modification involves alterations in structures such as penicillin-binding proteins or ribosomal sites, exemplified by PBP2a in methicillin-resistant *S. aureus* and D-Ala-D-Lac substitutions in vancomycin-resistant *Enterococcus faecium* and *Enterococcus faecalis* [7]. Reduced intracellular accumulation results from decreased membrane permeability due to porin loss and increased efflux activity, particularly RND-type pumps such as MexAB-OprM and AcrAB-TolC, which expel multiple antibiotic classes [7]. Biofilm formation further enhances resistance by limiting antibiotic penetration and promoting a metabolically dormant, drug-tolerant state. These mechanisms are frequently encoded on plasmids, transposons, or chromosomes, enabling horizontal gene transfer and rapid dissemination among pathogens [7].

According to Magiorakos et al. [8], bacteria are classified as multidrug-resistant (MDR), extensively drug-resistant (XDR), or pandrug-resistant (PDR). In addition, the Infectious Diseases Society of America and the Centers for Disease Control and Prevention highlight other clinically relevant resistant groups, including extended-spectrum cephalosporin-resistant (ESCR), carbapenem-resistant Enterobacteriaceae, difficult-to-treat (DTR) bacteria, carbapenem-resistant *A. baumannii*, and fluoroquinolone-resistant (FQR) organisms [9].

Given the substantial global burden of antimicrobial resistance, the particularly high projected burden in South Asia, where Pakistan is situated [1,3], and the prominent role of ESKAPEE pathogens in nosocomial infections, this study aims to determine the prevalence and antimicrobial resistance patterns of ESKAPEE pathogens in Pakistan, where comprehensive regional surveillance data on their distribution and resistance profiles remain limited. These findings are essential to inform infection-control strategies, antimicrobial stewardship, and clinical management in local health care settings.

## Methods

### Study setting

This descriptive cross-sectional study was conducted on clinical specimens collected from patients attending selected hospitals in Hyderabad, Sindh, Pakistan, including Taluka Hospitals in Latifabad (Shah Bhittai), Kotri, and Qasimabad, as well as Liaquat University Hospital. All microbiological processing, antimicrobial susceptibility testing, data compilation, and analysis were performed at the Department of Physiology, University of Sindh, Jamshoro.

The study was carried out over a 1-year period from August 2023 to August 2024. All laboratory records were anonymized, and no personal identifiers were included at any stage.

### Study population and sampling

The study included patients of all age groups, both outpatients and patients hospitalized for more than 48 hours, from major clinical units including the Medical, Surgical, Pediatric, Obstetrics and Gynecology departments; intensive care unit; and the cardiac care unit. Convenience sampling was employed by including all eligible clinical specimens received from the participating hospitals during the study period, based on their availability and predefined inclusion and exclusion criteria, without random selection.

### Inclusion and exclusion criteria

Patients with clinical features suggestive of urinary tract, upper or lower respiratory tract, and gastrointestinal tract infections were included, as well as those with sepsis, wound infections, abscesses, pus discharge, prolonged fever, or other clinically significant signs of infection. Patients who had received antimicrobial therapy within 2 weeks before specimen collection were excluded to reduce false-negative culture results due to suppressed bacterial growth.

### Specimen collection and transport

Clinical specimens included blood, urine, body fluids, sputum, tracheal aspirates, high vaginal swabs, pus, and wound swabs. Blood samples were collected aseptically and processed using the BacT/ALERT 3D automated blood culture system. Urine and other specimens were collected using standard aseptic techniques and transported in sterile containers. All procedures for specimen collection, handling, and transport followed Clinical and Laboratory Standards Institute (CLSI) guidelines (M40-A2 and GP41) [10,11].

### Laboratory methods

#### Culture isolation and identification

Positive blood cultures and other specimens were inoculated onto appropriate selective and differential media, including MacConkey agar, CLED agar, Mannitol Salt agar, Blood agar, and Salmonella-Shigella agar (Oxoid, Basingstoke, UK), according to specimen type, and incubated aerobically at 37°C for 24 hours. Bacterial identification was performed using Gram staining and conventional biochemical tests. Gram-positive organisms were identified by catalase and coagulase tests, while Gram-negative organisms were identified using standard biochemical reactions, including lactose fermentation, oxidase, citrate utilization, hydrogen sulfide production, Triple Sugar Iron test, motility, indole, urease, and Voges-Proskauer tests, following routine clinical microbiology protocols.

#### Antimicrobial susceptibility testing

Antimicrobial susceptibility testing was performed using the modified Kirby-Bauer disc diffusion method on Mueller-Hinton agar. Minimum inhibitory concentrations for selected antibiotics were determined by Mueller-Hinton broth dilution. Inoculum preparation, incubation, and interpretation were conducted according to CLSI guidelines (M100, 2023) and cross-checked with EUCAST 2023 breakpoints. Results were reported as susceptible, intermediate, or resistant based on standardized zone diameter and minimum inhibitory concentration criteria [12,13].

#### Resistance category definitions

Following the categorization of Magiorakos et al. [8], isolates showing resistance to at least one agent in three antimicrobial classes, in both Gram-positive and Gram-negative groups, were defined as multidrug-resistant (MDR). In *S. aureus*, MRSA isolates were classified as MDR due to resistance to oxacillin, penicillin, and cefoxitin, which indicates non-susceptibility to all β-lactam agents except anti-MRSA cephalosporins (ceftaroline/ceftobiprole), as per the MDR classification [8]. Extensively drug-resistant (XDR) was defined as resistance to one or more agents in all but two or fewer categories, whereas pandrug-resistant (PDR) was defined as resistance to all agents in all classes recommended by CLSI and EUCAST [8,12,13].

According to the Centers for Disease Control and Prevention (CDC), carbapenem resistance (CR) was defined as intermediate or resistant to one or more carbapenem agents in *P. aeruginosa* and *A. baumannii*, and resistant to *E. coli, Klebsiella* spp., and *Enterobacter* spp. (ertapenem only in Enterobacterales). ESCR included isolates resistant to one or more extended-spectrum cephalosporins (ceftazidime, cefepime, ceftriaxone, or cefotaxime). Fluoroquinolone resistance (FQR) was defined as resistance to one or more fluoroquinolones (ciprofloxacin, levofloxacin, or moxifloxacin) [14].

Difficult-to-treat resistance (DTR) applied to isolates that were intermediate or resistant to all tested agents within the following groups: carbapenems (imipenem, ertapenem, or meropenem; ertapenem only in Enterobacterales), β-lactams (ceftazidime, cefotaxime, cefepime, ceftriaxone, or piperacillin/tazobactam—excluding cefotaxime and ceftriaxone in *P. aeruginosa*), ampicillin/sulbactam (*A. baumannii* only), and fluoroquinolones (ciprofloxacin or levofloxacin) [9].

The term wild type was applied to isolates that did not exhibit any acquired resistance mechanisms phenotypically and were pansusceptible to tested agents, excluding those to which the species is intrinsically resistant [15]. In contrast, isolates resistant to all antibiotics tested in this study were designated as possible PDR. Since polymyxins and tigecycline were not included in the testing panel, the isolates could not be classified as true PDR but were designated as possible PDR according to standard definitions [8].

### Statistical analysis

The data were sorted and extracted using Microsoft Excel 2024, and were then analyzed using SPSS version 25.0 software (IBM) to determine the percentages, differences, and categorization of resistant isolates.

## Results

### Clinical isolates distribution

During the 2023-2024 study period, 1072 specimens were processed. Microbial growth was observed in 352 (32.8%) specimens, whereas 720 (67.2%) showed no growth. Among the positive cultures, ESKAPEE pathogens predominated (289, 82.1%), followed by other bacteria (27, 7.7%) and *Candida* species (36, 10.2%).

Within the ESKAPEE group, Enterobacterales were most common (187, 64.7%). *Escherichia coli* was the leading pathogen (144, 49.8%), followed by *Klebsiella pneumoniae* (38, 13.2%), *A. baumannii* (28, 9.7%), *P. aeruginosa* (27, 9.3%), *Enterococcus spp.* (24, 8.3%), *S. aureus* (23, 8.0%), and *Enterobacter* spp. (5, 1.7%).

### Specimen-wise distribution

Among the total specimens, midstream urine was the most frequent (680, 63.4%), followed by blood (247, 23.0%), pus (40, 3.7%), respiratory aspirates (34, 3.2%), sputum (33, 3.1%), and others (38, 3.6%).

From culture-positive samples, urine accounted for the majority (251, 71.3%), followed by blood (32, 9.1%), respiratory aspirates (25, 7.1%), pus (23, 6.5%), sputum (16, 4.6%), and others (5, 1.4%).

Most ESKAPEE pathogens were recovered from urine (201, 69.6%), followed by blood (25, 8.7%), respiratory aspirates (23, 7.6%), pus (22, 7.6%), sputum (15, 5.2%), and others (3, 1.0%).

### Demographics and specimen distribution of ESKAPEE isolates

The demographic characteristics, specimen sources, and organism distribution of patients with ESKAPEE isolates are summarized in Table 1. Among these patients, 172 (59.5%) were female and 117 (40.5%) were male, with most isolates recovered from inpatients (214, 74.0%) rather than outpatients (75, 26.0%).

**Table 1:** Demographic characteristics, specimen source, and organism profile of patients with ESKAPEE pathogen isolates (N = 289).

Category	Inpatients	Outpatients	Total
N (%)	214	75	289
**Gender**			
Male	90	27	117 (40.5%)
Female	124	48	172 (59.5%)
**Age group**			
0-9	22	0	22 (7.6%)
10-19	6	9	15 (5.2%)
20-29	18	17	35 (12.11%)
30-39	21	21	42 (14.5%)
40-49	22	6	28 (9.7%)
50-59	29	8	37 (12.8%)
60-69	28	9	37 (12.8%)
70-79	41	4	45 (15.6%)
80-89	27	1	28 (9.69%)
**Inpatient units**			
Medical ward	66 (30.8%)		214
ICU	47 (22.0%)		
Surgical ward	32 (15.0%)		
CCU	27 (12.6%)		
Pediatric ward	23 (10.7%)		
Obstetrics and gynecology	19 (8.9%)		
**Specimens**			
Urine	157	44	201 (69.6%)
Blood	17	8	25 (8.7%)
Pus	16	6	22 (7.6%)
Sputum	12	3	15 (5.1%)
Tracheal aspirate	12	11	23 (8%)
Others	0	3	3 (1%)
**Organism**			
*Escherichia coli*	109	35	144 (49.8%)
*Enterococcus* spp.	16	8	24 (8.3%)
*Staphylococcus aureus*	18	5	23 (8.0%)
*Klebsiella pneumoniae*	28	10	38 (13.1%)
*Acinetobacter baumannii*	19	9	28 (9.6%)
*Pseudomonas aeruginosa*	21	6	27 (9.3%)
*Enterobacter* spp.	3	2	5 (1.7%)

**Age-wise distribution** showed the highest proportion of ESKAPEE isolates among patients aged 70-79 years, followed by other older age groups, whereas patients aged 10-19 years accounted for the comparatively lowest proportion of isolates (Table 1).

Among hospitalized patients, isolates were most frequently recovered from the medical ward, followed by the intensive care unit (Table 1).

**Specimen-wise distribution** showed that *Escherichia coli* and *Enterococcus* spp. were predominantly isolated from urine specimens (125/144; 86.8% and 24/24; 100%, respectively). *K. pneumoniae* was mainly recovered from urine (21/38; 55.3%), followed by sputum (8/38; 21.1%) and respiratory aspirates (4/38; 10.5%). *S. aureus* was most frequently isolated from blood (13/23; 56.5%), while nonfermenters, including *A. baumannii* and *P. aeruginosa*, were primarily recovered from urine (11/28; 39.3% and 14/27; 51.9%) and respiratory specimens (7/28; 25.0% and 3/27; 11.1%). *Enterobacter* spp. was infrequently isolated, mainly from urine (3/5; 60%) and respiratory aspirates (1/5; 20%).

### ESKAPEE pathogens AMR profile

**Figure 1:**
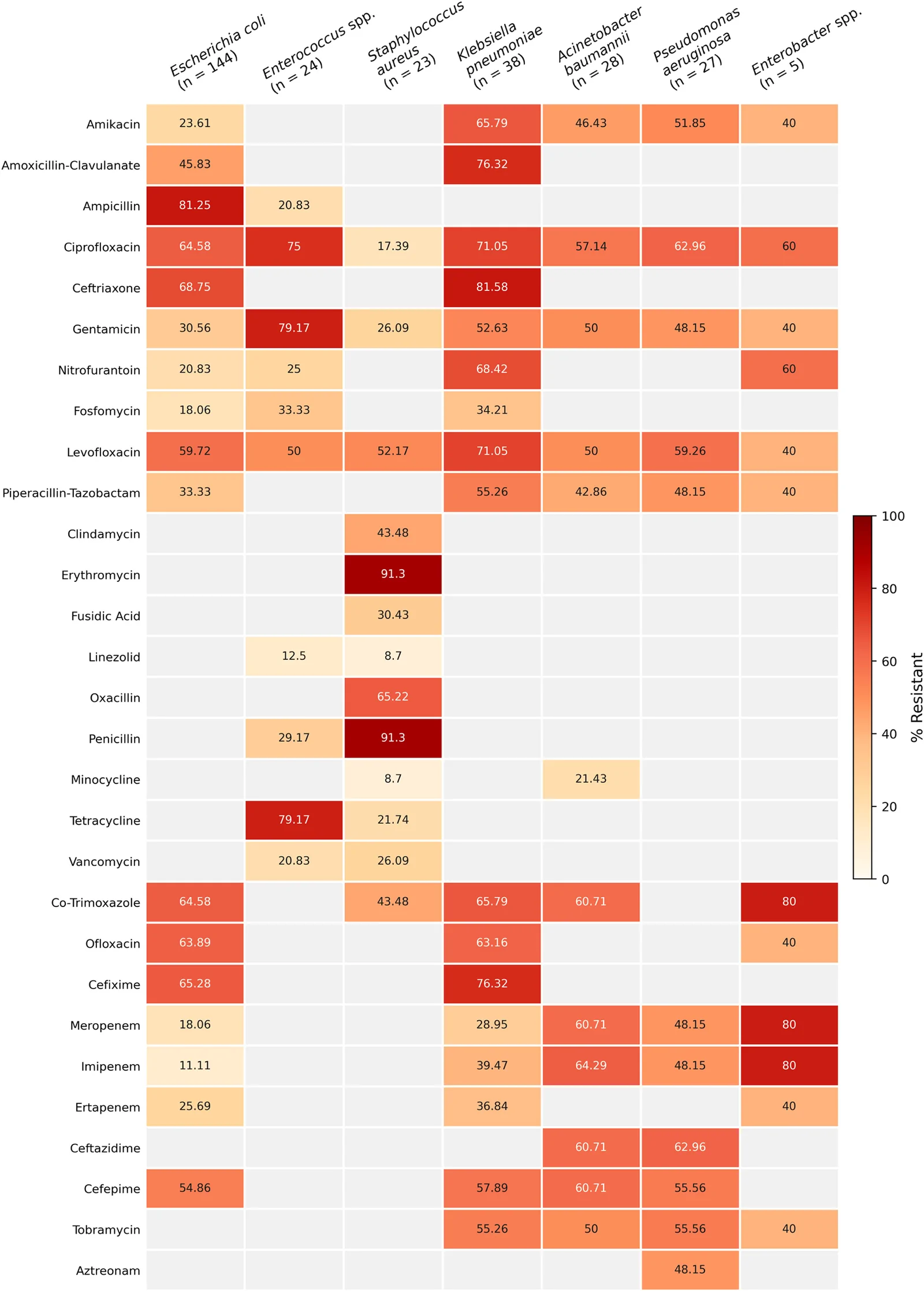
Heatmap of antimicrobial resistance (% resistant) among ESKAPEE pathogens isolated in this study. Color intensity reflects the percentage of isolates resistant to each antibiotic, from pale orange (low resistance) to dark red (high resistance), as indicated by the color scale. Values within each cell denote the percentage of resistant isolates. Gray cells indicate antibiotic–organism combinations that were not tested or were not clinically applicable. **^a^** Gentamicin resistance for *Enterococcus* spp. reflects high-level aminoglycoside resistance (HLAR) screening performed to assess potential synergy with a cell wall–active agent, rather than standalone susceptibility testing. **^b^** Fosfomycin susceptibility for *K pneumoniae* is reported for informational purposes only; its clinical efficacy against this organism is not well established, and it is not recommended as a primary treatment option. Full susceptible/intermediate/resistant counts and percentages for all antibiotic–organism combinations are provided in Supplementary Table S1.

The antimicrobial resistance patterns of ESKAPEE pathogens are illustrated in Figure 1. Among Gram-positive isolates, *S. aureus* showed high resistance to erythromycin and penicillin (91.30% each), while *Enterococcus* spp. showed high resistance to gentamicin (79.17%, high-level aminoglycoside resistance) and tetracycline (79.17%). Vancomycin and linezolid retained the greatest activity against both organisms.

Among Gram-negative isolates, resistance to third-generation cephalosporins and ciprofloxacin/ofloxacin exceeded 60% in most organisms, with slightly lower resistance to levofloxacin. Carbapenem resistance was lowest in *E. coli* (11.11-25.69%) but remained substantial in *K. pneumoniae* and *P. aeruginosa*, with the highest levels among adequately sampled organisms observed in *A. baumannii* (60.71-64.29%). *Enterobacter* spp. showed an even higher carbapenem resistance rate (80% for meropenem and imipenem); however, this finding should be interpreted with caution because only five isolates were recovered. Full susceptibility data for all organism–antibiotic combinations are provided in Supplementary Table S1.

### AMR categories

**Figure 2:**
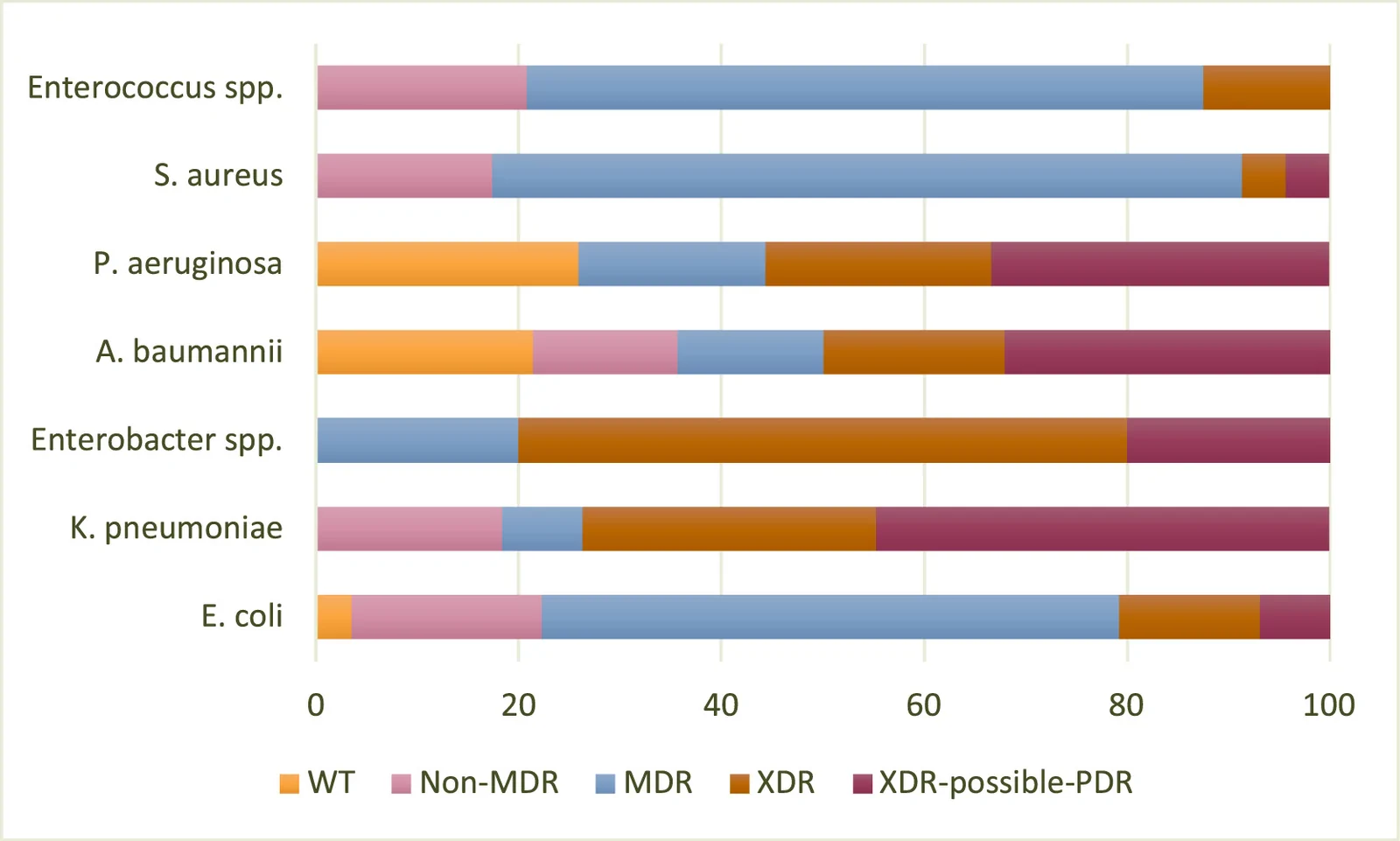
ESKAPEE isolates resistance categories, Magiorakos et al. [8] classification. Abbreviations: MDR, multidrug-resistant; PDR, pandrug-resistant; WT, wild type; XDR, extensively drug-resistant.

**Figure 3:**
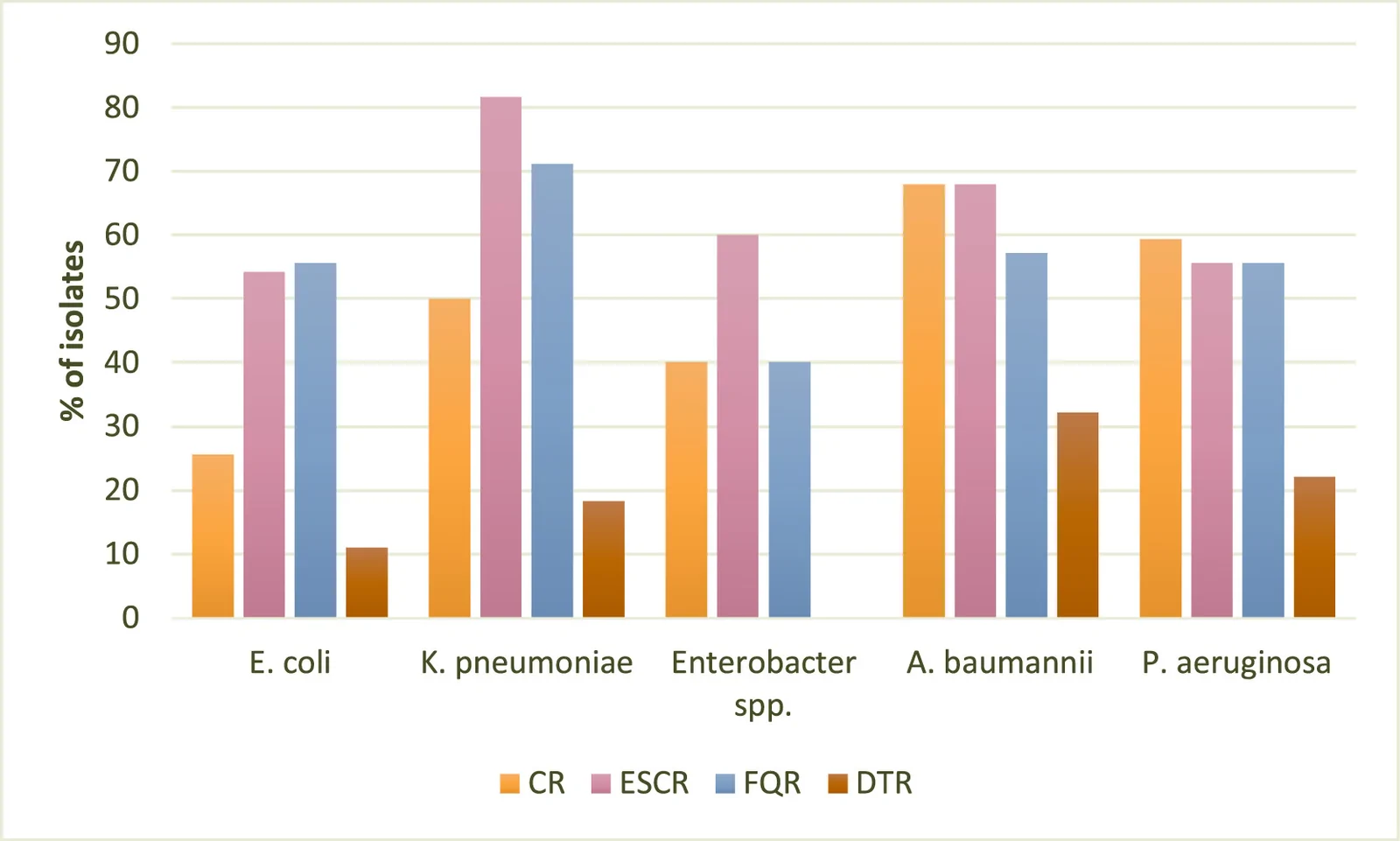
Distribution of CDC and IDSA resistance phenotypes [9] among Gram-negative ESKAPEE pathogens. Abbreviations: CDC, Centers for Disease Control and Prevention; CR, carbapenem-resistant; DTR, difficult-to-treat resistance; ESCR, extended-spectrum cephalosporin-resistant; FQR, fluoroquinolone-resistant; IDSA, Infectious Diseases Society of America.

ESKAPEE pathogens were classified according to international resistance criteria [8,9], with detailed distributions shown in Table 2 and illustrated in Figure 2, Figure 3.

**Table 2:** Comparative classification of ESKAPEE pathogens based on international drug resistance criteria [8,9].

Microorganism		Drug Resistance Classification [8] No. (%)					CDC & IDSA Phenotypes [9] No. (%)			
	Total (*n*)	WT	Non-MDR	MDR	XDR	Possible PDR	CR	ESCR	FQR	DTR
***E coli***	144	5 (3.5)	27 (18.8)	82 (56.9)	20 (13.9)	10 (6.9)	37 (25.7)	78 (54.2)	80 (55.6)	16 (11.1)
***K pneumoniae***	38	0	7 (18.4)	3 (7.9)	11 (28.9)	17 (44.7)	19 (50)	31 (81.6)	27 (71.1)	7 (18.4)
***Enterobacter spp.***	5	0	0	1 (20)	3 (60)	1 (20)	2 (40)	3 (60)	2 (40)	0
***A baumannii***	28	6 (21.4)	4 (14.3)	4 (14.3)	5 (17.9)	9 (32.1)	19 (67.9)	19 (67.9)	16 (57.1)	9 (32.1)
***P aeruginosa***	27	7 (25.9)	0	5 (18.5)	6 (22.2)	9 (33.3)	16 (59.3)	15 (55.6)	15 (55.6)	6 (22.2)
***S aureus***	23	0	4 (17.4)	17 (73.9)	1 (4.3)	1 (4.3)	NA	NA	NA	NA
***Enterococcus spp.***	24	0	5 (20.8)	16 (66.7)	3 (12.5)	0	NA	NA	NA	NA

Abbreviations: CDC, Centers for Disease Control and Prevention; CR, carbapenem-resistant; DTR, difficult-to-treat resistance; ESCR, extended-spectrum cephalosporin-resistant; FQR, fluoroquinolone-resistant; IDSA, Infectious Diseases Society of America; MDR, multidrug-resistant; NA, not applicable; PDR, pandrug-resistant; WT, wild type; XDR, extensively drug-resistant.

Among Gram-negative isolates, MDR was the predominant phenotype in *E. coli* (56.9%), whereas XDR and possible PDR phenotypes were more frequent among *K. pneumoniae, A. baumannii, P. aeruginosa*, and *Enterobacter* spp. (Figure 2). Carbapenem resistance was lowest in *E. coli* (25.7%) and highest in *A. baumannii* (67.9%), with intermediate rates observed in *K pneumoniae, Enterobacter* spp., and *P. aeruginosa*. ESCR exceeded 50% across all Gram-negative organisms, whereas FQR exceeded 50% in all except *Enterobacter* spp. (40%) (Figure 3).

Among Gram-positive isolates, MDR predominated in both *S. aureus* (73.9%) and *Enterococcus* spp. (66.7%), with XDR and possible PDR phenotypes comparatively rare (Figure 2); CDC/Infectious Diseases Society of America (IDSA) phenotyping criteria [9] were not applicable to these organisms. No isolate could be classified as true PDR [8], as the complete CLSI/EUCAST-recommended antibiotic panels were not tested [12,13].

## Discussion

This study provides a comprehensive overview of antimicrobial resistance trends among ESKAPEE pathogens isolated from diverse clinical specimens in Hyderabad, Pakistan. Although *Escherichia coli* is not formally classified within the ESKAPEE group, it was the predominant pathogen in this study, followed by *K. pneumoniae, A. baumannii, P. aeruginosa, Enterococcus* spp., *S. aureus*, and *Enterobacter* spp. These organisms are well recognized for their ability to acquire and disseminate resistance determinants, facilitating evasion of antimicrobial therapy [[16], [17], [18]]. The ESKAPEE pathogens were designated by the IDSA as major contributors to health care-associated infections [17] and were subsequently included among the World Health Organization priority pathogens requiring urgent antibiotic development [18]. Consistent with reports from Europe documenting high levels of resistance in *E. coli*, particularly to β-lactams, fluoroquinolones, aminoglycosides, and sulfonamides [18], our findings similarly demonstrate the predominance of *E. coli* in the local clinical setting.

***E. coli*** exhibited high resistance to ampicillin (81.2%) and third-generation cephalosporins (68.7%), consistent with reports from South and Southeast Asia [19]. Carbapenems retained partial activity, with 11% to 18% resistance to imipenem and meropenem, while 25.7% of isolates were resistant to ertapenem, indicating early erosion of last-resort options. Based on Magiorakos et al. [8] and IDSA [9] criteria, 56.9% of *E. coli* isolates were MDR, 13.9% XDR, and 6.9% possible PDR, with CR, ESCR, FQR, and DTR rates of 25.7%, 54.2%, 55.6%, and 11.1%, respectively. These findings align with regional studies, including Hashem et al. [20] from Iraq, Camacho-Ortiz et al. [14] from Mexico, and Gondal et al. [21] from Pakistan, underscoring the persistent burden of multidrug and extended-spectrum resistance in *E. coli* and highlighting the emerging threat of carbapenem and fluoroquinolone resistance to empirical therapy and infection control.

***K. pneumoniae*** demonstrated high resistance rates, particularly to β-lactam/β-lactamase inhibitor combinations (76.3%) and cephalosporins (81.6%), consistent with reports from Eastern India and the Middle East [22,23]. MDR, XDR, possible PDR, and DTR rates were 7.9%, 28.9%, 44.7%, and 18.4%, respectively, indicating a predominance of XDR and possible PDR strains. The XDR prevalence was comparable to that reported in Greece (26.8%) [24], while the MDR rate was lower than that reported from southeastern Romania (24.4%) [25]. Resistance phenotypes including CR (50%), ESCR (81.6%), FQR (71.1%), and DTR (18.4%) partially align with findings from China [25], while the FQR rate is similar to that reported in a recent study from Hyderabad (64.29%) [26]. Although CR rates were broadly consistent, higher ESCR and FQR rates and lower DTR rates in this study highlight regional differences in resistance dynamics. These findings underscore the global dissemination of ESBL- and carbapenemase-producing *K pneumoniae*, with concurrent ESCR and FQR phenotypes supporting plasmid-mediated spread of resistance determinants such as *bla*CTX-M, *bla*KPC, and *qnr* genes [27].

***Enterobacter spp.*** were isolated in low numbers but displayed MDR (20%), XDR (60%), and possible PDR (20%) phenotypes, along with CR (40%), ESCR (60%), and FQR (40%). Comparable trends have been reported from a hospital in Mexico, with MDR (26.6%), ESCR (45.2%), and FQR (21.5%), although CR and DTR rates were notably lower (5.1% and 2.3%) [14], likely reflecting sample size or regional variation. No DTR isolates were identified in the present study. Despite the limited number of isolates, these findings indicate the potential emergence of highly resistant *Enterobacter* strains in clinical settings.

***A. baumannii*** exhibited extensive resistance, with CR and ESCR each at 67.9%, FQR at 57.1%, and the highest DTR rate (32.1%) among ESKAPEE isolates. MDR, XDR, and possible PDR rates were 14.3%, 17.9%, and 32.1%, respectively, consistent with reports from Greece (XDR/PDR 34.3%) [24] and China (CR, ESCR, FQR, and DTR >80%) [28].

***P. aeruginosa*** showed CR, ESCR, and FQR >50%, with DTR at 22.2%, and MDR, XDR, and possible PDR rates of 18.5%, 22.2%, and 33.3%, respectively, comparable to reports from Mexico, Lebanon, and Iran [14,29,30]. DTR and CR rates in Lebanon were 30% to 50% lower than those observed here, reflecting geographic variation and differences in nosocomial resistance gene prevalence. These findings align with global data identifying carbapenem-resistant *Acinetobacter* and *Pseudomonas* as major nosocomial pathogens, often associated with intensive care unit and ventilator-related infections, driven by antibiotic selection pressure and broad-spectrum agent use [31,32]. The emergence of possible PDR isolates among these species underscores their significant clinical threat, particularly in critical care settings.

***S. aureus*** showed 65.2% resistance to oxacillin, indicating a high MRSA prevalence, along with 91.3% resistance to erythromycin and penicillin. MDR, XDR, and possible PDR rates were 73.9%, 4.3%, and 4.3%, respectively. Similar findings have been reported by Pandey et al. [33] from Nepal, with MRSA at 58%, MDR at 68.2%, and erythromycin resistance at 75%. Linezolid and vancomycin remained largely effective in the present study (91.3% and 73.9%), consistent with those reported by Pandey et al. [33], confirming their continued reliability against MRSA infections. MRSA-associated XDR prevalence reported in Indonesia and India ranged from 0.77% to 15.1%, supporting the XDR rate observed here [34,35] and reflecting regional variability likely driven by antibiotic overuse and inadequate antimicrobial stewardship.

***Enterococcus spp.*** exhibited MDR in 66.7% and XDR in 12.5% of isolates, with vancomycin and linezolid resistance at 20.8% and 12.5%, respectively. These trends are comparable to findings from India, where MDR and XDR rates showed an inverse pattern (28.9% and 35.6%) [35], likely reflecting differences in VRE gene distribution. Vancomycin resistance was also reported at 20% by Pandey et al. [33], supporting regional patterns of VRE prevalence in *E faecium* across Asian clinical centers. The presence of VRE highlights the need for continued surveillance, given its potential as a reservoir for transmissible resistance genes.

ESKAPEE pathogens were most frequently recovered from urine samples (69.6%), particularly among females (34.3%) and patients over 65 years of age (46%). Similar patterns were observed in India, where urine was the predominant source and recovery was higher in females (55.1%) and older patients (>61 years, 16.4%) [33]. These findings indicate increased susceptibility in the elderly, likely due to comorbidities and greater antibiotic exposure, and highlight the urinary tract as a major reservoir for resistant Enterobacterales, facilitating potential transmission from the community to hospital settings.

The study underscores multiple drivers of escalating antimicrobial resistance in Pakistan, including unregulated antibiotic access, empirical prescribing, limited diagnostic capacity, and inadequate infection control. Previous studies have reported widespread dispensing of antibiotics without a prescription in community pharmacies across Pakistan. In one survey, 67% of individuals obtained antibiotics without a physician's prescription, highlighting weak regulatory enforcement and inappropriate antibiotic use [36]. Qualitative evidence from Pakistani migrants in New Zealand also indicates that antibiotics purchased in Pakistan are frequently carried abroad for subsequent self-medication, further illustrating their easy availability without prescription [37]. These practices collectively contribute to inappropriate antimicrobial use and increase the selection pressure for resistant organisms.

The rising prevalence of carbapenem resistance and possible PDR [8] among ESKAPEE pathogens emphasizes the need for urgent national and institutional interventions. These findings reflect regional and global resistance trends and call for integrated strategies combining molecular and epidemiological surveillance, antimicrobial stewardship, and public health education. Strengthening national AMR frameworks and enforcing rational antibiotic use are critical to preserving the efficacy of last-resort antimicrobials.

## Conclusion and future directions

The study demonstrates a high prevalence of multidrug- and extensively drug-resistant [8] ESKAPEE pathogens in Hyderabad clinical settings, posing a significant threat to treatment efficacy and infection-control programs. Among Gram-negative isolates, *E. coli* and *K. pneumoniae* were the predominant MDR and ESCR producers, while *A. baumannii* and *P. aeruginosa* showed substantial carbapenem resistance, reflecting declining effectiveness of frontline and last-line antibiotics. Gram-positive pathogens, including MRSA and VRE, further challenge clinical management, although linezolid and vancomycin largely retained activity. The predominance of urinary isolates highlights urinary tract infections as the principal source of multidrug-resistant ESKAPEE isolates in this study, suggesting that reducing urinary tract infections could substantially lessen the overall burden of antimicrobial resistance.

These findings emphasize the urgent need for robust antimicrobial stewardship, continuous susceptibility surveillance, and stringent infection-control measures. Targeted preventive strategies, particularly among older adults, who represented the largest proportion of cases in this study, may help reduce this burden before antibiotic treatment becomes necessary. In line with IDSA and CDC recommendations for catheter-associated urinary tract infection prevention [38], these include minimizing unnecessary or prolonged urinary catheterization, aseptic catheter insertion and care, improved personal hygiene, appropriate diagnosis and prompt treatment of symptomatic urinary tract infections, and avoidance of antimicrobial prophylaxis for asymptomatic bacteriuria. Evaluating the specific effectiveness of such interventions within Pakistan's health care context falls outside the scope of the present study and warrants dedicated future research.

Future research should focus on molecular characterization of resistance determinants, plasmid profiling, and genomic mapping to clarify transmission dynamics. Investigating novel therapeutic combinations and re-evaluating older agents such as fosfomycin or minocycline may offer interim treatment options. National surveillance programs, enhanced clinician awareness, and targeted public health initiatives are critical to mitigating this escalating antimicrobial resistance crisis in Pakistan.

## CRediT authorship contribution statement

**Muhammad Aqif Ilyas:** Conceptualization, Data curation, Formal analysis, Investigation, Methodology, Project administration, Resources, Software, Supervision, Validation, Visualization, Writing – original draft, Writing – review & editing. **Muhammad Sameer Hashmi:** Formal analysis, Investigation, Methodology, Validation, Visualization, Writing – review & editing.

## Declaration of competing interest

The authors declare no competing interests.

## Appendix Supplementary materials

Image, application 1

## References

[1] Collaborators Antimicrobial Resistance (2022). Global burden of bacterial antimicrobial resistance in 2019: a systematic analysis. Lancet.

[2] O’Neill J. (2016). Tackling Drug-Resistant Infections Globally: Final Report and Recommendations. Review on Antimicrobial Resistance. Wellcome Trust and HM Government.

[3] GBD 2021 Antimicrobial Resistance Collaborators (2024). Global burden of bacterial antimicrobial resistance 1990–2021: a systematic analysis with forecasts to 2050. Lancet.

[4] Mulani M.S., Kamble E.E., Kumkar S.N., Tawre M.S., Pardesi KR. (2019). Emerging strategies to combat ESKAPE pathogens in the era of antimicrobial resistance: a review. Front Microbiol.

[5] Mahmood H.Y., Jamshidi S., Sutton J.M., Rahman KM. (2016). Current advances in developing inhibitors of bacterial multidrug efflux pumps. Curr Med Chem.

[6] Rice LB. (2008). Federal funding for the study of antimicrobial resistance in nosocomial pathogens: no ESKAPE. J Infect Dis.

[7] Santajit S., Indrawattana N. (2016). Mechanisms of antimicrobial resistance in ESKAPE pathogens. BioMed Res Int.

[8] Magiorakos A.P., Srinivasan A., Carey R.B., Carmeli Y., Falagas M.E., Giske C.G. (2012). Multidrug-resistant, extensively drug-resistant and pandrug-resistant bacteria: an international expert proposal for interim standard definitions for acquired resistance. Clin Microbiol Infect.

[9] Kadri S.S., Adjemian J., Lai Y.L., Spaulding A.B., Ricotta E., Prevots D.R. (2018). Difficult-to-treat resistance in Gram-negative bacteremia at 173 US hospitals: retrospective cohort analysis of prevalence, predictors, and outcome of resistance to all first-line agents. Clin Infect Dis.

[10] Clinical, Laboratory (2017). https://clsi.org/shop/standards/pre02/.

[11] Clinical, Laboratory (2014). https://clsi.org/shop/standards/m40/.

[12] Clinical, institute LS. Performance standards for antimicrobial susceptibility testing. Wayne, PA: Clinical and Laboratory Standards Institute; 2023. https://clsi.org/shop/standards/m100/.

[13] The European committee on antimicrobial susceptibility T. Breakpoint tables for interpretation of MICs and zone diameters. version 13.0; 2023. https://www.eucast.org/fileadmin/src/media/PDFs/EUCAST_files/Breakpoint_tables/v_13.0_Breakpoint_Tables.pdf [accessed 8 August 2026].

[14] Camacho-Ortiz A., Flores-Treviño S., Bocanegra-Ibarias P. (2025). Prevalence of difficult-to-treat resistance in ESKAPE pathogens in a third level hospital in Mexico. Infect Prev Pract.

[15] The European committee on antimicrobial susceptibility T. Implications of breakpoints splitting the wild type and/or resistant populations. Basel, Switzerland: EUCAST; 2016. https://www.eucast.org/clinical_breakpoints_and_dosing/splitting_mic_wild_type_distributions/ [accessed 8 August 2026].

[16] Miller W.R., Arias CAO. (2024). ESKAPE pathogens: antimicrobial resistance, epidemiology, clinical impact and therapeutics. Nat Rev Microbiol.

[17] Li Z., Xie J., Yang J.A.O., Liu S., Ding Z., Hao J. (2021). Pathogenic characteristics and risk factors for ESKAPE pathogens infection in burn patients. Infect Drug Resist.

[18] De Oliveira D.M.P., Forde B.M., Kidd T.J., Harris P.N.A., Schembri M.A., Beatson S.A., Paterson D.L., Walker M.J. (2020). Antimicrobial Resistance in ESKAPE Pathogens. Clin Microbiol Rev.

[19] Duguid R.C., Ashley E.A.O., Turner P.A.O., Douangnouvong A., Panyaviseth P., Wijeratne P. (2025). Antimicrobial resistance among children in Southeast Asia: a systematic review. BMJ Public Health.

[20] Hashem R.M., Ali M.M., Al-Hasnawi A.T.N. (2024). Unveiling ESBL-Producing E. coli: genetic Markers, antibiotic Resistance, and Virulence Factors in UTI Patients Exhibiting MDR, XDR, and PDR phenotypic bacterial isolates. Pak.j.life.soc.sci.

[21] Gondal A.J., Choudhry N., Bukhari H., Rizvi Z., Jahan S., Yasmin N. (2023). Estimation, evaluation and characterization of carbapenem resistance burden from a tertiary Care Hospital. Pakistan. Antibiotics (Basel).

[22] Sau B., Ghosh S., Mal S., Adhikary P. (2025). Bacteriological profile and antimicrobial resistance patterns in neonatal sepsis: special reference to Klebsiella pneumoniae at a tertiary Care Hospital in Eastern India. Cureus.

[23] Al-Zalabani A, AlThobyane OA, Alshehri AH, Alrehaili AO, Namankani MO, Aljafri OH Prevalence of Klebsiella pneumoniae antibiotic resistance in Medina, Saudi Arabia, 2014–2018. https://www.cureus.com/articles/38260-prevalence-of-klebsiella-pneumoniae-antibiotic-resistance-in-medina-saudi-arabia-2014-2018. [accessed 8 August 2026]. 10.7759/cureus.9714 PMC7489314 32944436

[24] Lagadinou M., Amerali M., Michailides C., Chondroleou A., Skintzi K., Spiliopoulou A. (2024). Antibiotic resistance trends in carbapenem-resistant Gram-negative pathogens and eight-year surveillance of XDR bloodstream infections in a Western Greece tertiary hospital. Pathogens.

[25] Arbune M., Gurau G., Niculet E., Iancu A.V., Lupasteanu G., Fotea S. (2021). Prevalence of antibiotic resistance of ESKAPE pathogens over five years in an infectious diseases hospital from South-East of Romania. Infect Drug Resist.

[26] Ilyas M.A., Bano S., Tunio S.A., Mangi A.A., Detho H. (2025). Frequency of carbapenem resistance in the pathogenic Gram-negative bacteria from Hyderabad, Sindh: carbapenem resistance in the pathogenic Gram-negative bacteria. PJHS-Lahore.

[27] Idrees E.K., Aldriwesh M.G., Alkhulaifi M.M., Alghoribi MF. (2025). Systematic review of multidrug-resistant Klebsiella pneumoniae in the Arabian Peninsula: molecular epidemiology and resistance patterns. Front Microbiol.

[28] Zhang Z., Tian L. (2022). Trends in DTR, CR, ECR, and FQR in four common Gram-negative bacteria: a retrospective study from 2013 to 2021. Infect Drug Resist.

[29] Itani R., Khojah H.M.J., Mukattash T.L., Shuhaiber P., Raychouni H., Dib C. (2025). Difficult-to-treat resistant Pseudomonas aeruginosa infections in Lebanese hospitals: impact on mortality and the role of initial antibiotic therapy. PLoS One.

[30] Saderi H., Owlia P. (2015). Detection of multidrug resistant (MDR) and extremely drug resistant (XDR) P. aeruginosa isolated from patients in Tehran, Iran. Iran J Pathol.

[31] Boutzoukas A., Doi Y. (2025). The global epidemiology of carbapenem-resistant Acinetobacter baumannii. JAC Antimicrob Resist.

[32] Saravanan M., Belete M.A.O., Arockiaraj JAO. (2023). Carbapenem-resistant Pseudomonas aeruginosa in intensive care units increase mortality as an emerging global threat. Int J Surg.

[33] Pandey R., Mishra S.K., Shrestha A. (2021). Characterisation of ESKAPE pathogens with special reference to multidrug resistance and biofilm production in a Nepalese hospital. Infect Drug Resist.

[34] Haliman C.C., Prasetyo D.S., Tjampakasari C.R., Ibrahim F., Sudiro TM. (2023). Multidrug resistance and extensively drug-resistance in Staphylococcus aureus, Staphylococcus epidermidis, and Staphylococcus haemolyticus. Microbiol Indones.

[35] Basak S., Singh P., Rajurkar M. (2016). Multidrug resistant and extensively drug resistant bacteria: a study. J Pathog.

[36] Nohri A.R., Siddiqui M.I., Usman G., Sarang S., Memon H.Q., Singh D. (2024). Antibiotic dispensation without prescription by community pharmacies in Pakistan. J Med Surg Public Health.

[37] Akhtar S.S., Heydon S., Norris P. (2022). Bringing medicine from Pakistan and self-medication among Pakistani mothers in New Zealand. J Immigr Minor Health.

[38] Patel P.K., Advani S.D., Kofman A.D., Lo E., Maragakis L.L., Pegues D.A. (2023). Strategies to prevent catheter-associated urinary tract infections in acute-care hospitals: 2022 update. Infect Control Hosp Epidemiol.

