# Supplementary material for: Prevalence and antimicrobial resistance trends of ESKAPEE pathogens in clinical isolates from Hyderabad, Pakistan: a cross-sectional study
Source: IJID Reg. 2026 Jul 18;20:100950. doi: 10.1016/j.ijregi.2026.100950 (PMC13499059; doi:10.1016/j.ijregi.2026.100950)
Supplement: Supplementary file 1 [file mmc1.docx]

**Supplementary Data**

**Table S1**. Antimicrobial susceptibility profile of ESKAPE pathogens by disc diffusion and MIC.

| **Organism** | ***Escherichia coli* ( n = 144 )** | | ***Enterococcus Spp.* ( n = 24 )** | | ***Staphylococcus aureus* ( n = 23 )** | | ***Klebsiella pneumoniae* ( n = 38 )** | | ***Acinetobacter baumannii*  ( n = 28 )** | | ***Pseudomonas aeruginosa* ( n = 27 )** | | ***Enterobacter Spp.* (n = 5)** | |
| --- | --- | --- | --- | --- | --- | --- | --- | --- | --- | --- | --- | --- | --- | --- |
| **Antibiotics** | **S %** | **R %** | **S %** | **R %** | **S %** | **R %** | **S %** | **R %** | **S %** | **R %** | **S %** | **R %** | **S %** | **R %** |
| **Amikacin** | 110 (76.39%) | 34 (23.61%) | - | - | - | - | 13 (34.21%) | 25 (65.79%) | 15 (53.57%) | 13 (46.43%) | 13 (48.15%) | 14 (51.85%) | 3 (60.00%) | 2 (40.00%) |
| **Amoxicillin – Clavulanate** | 78 (54.17%) | 66 (45.83%) | NA | NA | - | - | 9 (23.68%) | 29 (76.32%) | NA | NA | NA | NA | NA | NA |
| **Ampicillin** | 27 (18.75%) | 117 (81.25%) | 19 (79.17%) | 5 (20.83%) | NA | NA | NA | NA | NA | NA | NA | NA | NA | NA |
| **Ciprofloxacin** | 51 (35.42%) | 93 (64.58%) | 6 (25.00%) | 18 (75.00%) | 19 (82.61%) | 4 (17.39%) | 11 (28.95%) | 27 (71.05%) | 12 (42.86%) | 16 (57.14%) | 10 (37.04%) | 17 (62.96%) | 2 (40.00%) | 3 (60.00%) |
| **Ceftriaxone** | 45 (31.25%) | 99 (68.75%) | NA | NA | NA | NA | 7 (18.42%) | 31 (81.58%) | NA | NA | NA | NA | NA | NA |
| **Gentamicin** | 100 (69.44%) | 44 (30.56%) | 5 (20.83%) ^a^ | 19 (79.17%) ^a^ | 17 (73.91%) | 6 (26.09%) | 18 (47.37%) | 20 (52.63%) | 14 (50.00%) | 14 (50.00%) | 14 (51.85%) | 13 (48.15%) | 3 (60.00%) | 2 (40.00%) |
| **Nitrofurantoin** | 114 (79.17%) | 30 (20.83%) | 18 (75.00%) | 6 (25.00%) | - | - | 12 (31.58%) | 26 (68.42%) | - | - | - | - | 2 (40.00%) | 3 (60.00%) |
| **Fosfomycin** | 118 (81.94%) | 26 (18.06%) | 16 (66.67%) | 8 (33.33%) | - | - | 25 (65.79%) ^b^ | 13 (34.21%) ^b^ | NA | NA | NA | NA | NA | NA |
| **Levofloxacin** | 58 (40.28%) | 86 (59.72%) | 12 (50.00%) | 12 (50.00%) | 11 (47.83%) | 12 (52.17%) | 11 (28.95%) | 27 (71.05%) | 14 (50.00%) | 14 (50.00%) | 11 (40.74%) | 16 (59.26%) | 3 (60.00%) | 2 (40.00%) |
| **Piperacillin – Tazobactam** | 96 (66.67%) | 48 (33.33%) | NA | NA | - | - | 17 (44.74%) | 21 (55.26%) | 16 (57.14%) | 12 (42.86%) | 14 (51.85%) | 13 (48.15%) | 3 (60.00%) | 2 (40.00%) |
| **Clindamycin** | NA | NA | NA | NA | 13 (56.52%) | 10 (43.48%) | NA | NA | NA | NA | NA | NA | NA | NA |
| **Erythromycin** | NA | NA | - | - | 2 (8.70%) | 21 (91.30%) | NA | NA | NA | NA | NA | NA | NA | NA |
| **Fusidic Acid** | NA | NA | NA | NA | 16 (69.57%) | 7 (30.43%) | NA | NA | NA | NA | NA | NA | NA | NA |
| **Linezolid** | NA | NA | 21 (87.50%) | 3 (12.50%) | 21 (91.30%) | 2 (8.70%) | NA | NA | NA | NA | NA | NA | NA | NA |
| **Oxacillin** | NA | NA | NA | NA | 8 (34.78%) | 15 (65.22%) | NA | NA | NA | NA | NA | NA | NA | NA |
| **Penicillin** | NA | NA | 17 (70.83%) | 7 (29.17%) | 2 (8.70%) | 21 (91.30%) | NA | NA | NA | NA | NA | NA | NA | NA |
| **Minocycline** | - | - | - | - | 21 (91.30%) | 2 (8.70%) | - | - | 22 (78.57%) | 6 (21.43%) | NA | NA | - | - |
| **Tetracycline** | - | - | 5 (20.83%) | 19 (79.17%) | 18 (78.26%) | 5 (21.74%) | - | - | - | - | NA | NA | - | - |
| **Vancomycin** | NA | NA | 19 (79.17%) | 5 (20.83%) | 17 (73.91%) | 6 (26.09%) | NA | NA | NA | NA | NA | NA | NA | NA |
| **Co – Trimoxazole** | 51 (35.42%) | 93 (64.58%) | NA | NA | 13 (56.52%) | 10 (43.48%) | 13 (34.21%) | 25 (65.79%) | 11 (39.29%) | 17 (60.71%) | NA | NA | 1 (20.00%) | 4 (80.00%) |
| **Ofloxacin** | 52 (36.11%) | 92 (63.89%) | NA | NA | - | - | 14 (36.84%) | 24 (63.16%) | - | - | - | - | 3 (60.00%) | 2 (40.00%) |
| **Cefixime** | 50 (34.72%) | 94 (65.28%) | NA | NA | NA | NA | 9 (23.68%) | 29 (76.32%) | NA | NA | NA | NA | NA | NA |
| **Meropenem** | 118 (81.94%) | 26 (18.06%) | NA | NA | NA | NA | 27 (71.05%) | 11 (28.95%) | 11 (39.29%) | 17 (60.71%) | 14 (51.85%) | 13 (48.15%) | 1 (20.00%) | 4 (80.00%) |
| **Imipenem** | 128 (88.89%) | 16 (11.11%) | NA | NA | NA | NA | 23 (60.53%) | 15 (39.47%) | 10 (35.71%) | 18 (64.29%) | 14 (51.85%) | 13 (48.15%) | 1 (20.00%) | 4 (80.00%) |
| **Ertapenem** | 107 (74.31%) | 37 (25.69%) | NA | NA | NA | NA | 24 (63.16%) | 14 (36.84%) | NA | NA | NA | NA | 3 (60.00%) | 2 (40.00%) |
| **Ceftazidime** | - | - | NA | NA | NA | NA | - | - | 11 (39.29%) | 17 (60.71%) | 10 (37.04%) | 17 (62.96%) | - | - |
| **Cefepime** | 65 (45.14%) | 79 (54.86%) | NA | NA | NA | NA | 16 (42.11%) | 22 (57.89%) | 11 (39.29%) | 17 (60.71%) | 12 (44.44%) | 15 (55.56%) | - | - |
| **Tobramycin** | - | - | - | - | - | - | 17 (44.74%) | 21 (55.26%) | 14 (50.00%) | 14 (50.00%) | 12 (44.44%) | 15 (55.56%) | 3 (60.00%) | 2 (40.00%) |
| **Aztreonam** | - | - | NA | NA | NA | NA | - | - | NA | NA | 14 (51.85%) | 13 (48.15%) | - | - |
